# Neutrophil Responses to Sterile Implant Materials

**DOI:** 10.1371/journal.pone.0137550

**Published:** 2015-09-10

**Authors:** Siddharth Jhunjhunwala, Stephanie Aresta-DaSilva, Katherine Tang, David Alvarez, Matthew J. Webber, Benjamin C. Tang, Danya M. Lavin, Omid Veiseh, Joshua C. Doloff, Suman Bose, Arturo Vegas, Minglin Ma, Gaurav Sahay, Alan Chiu, Andrew Bader, Erin Langan, Sean Siebert, Jie Li, Dale L. Greiner, Peter E. Newburger, Ulrich H. von Andrian, Robert Langer, Daniel G. Anderson

**Affiliations:** 1 David H. Koch Institute for Integrative Cancer Research, Massachusetts Institute of Technology, Cambridge, Massachusetts, 02139, United States of America; 2 Department of Chemical Engineering, Massachusetts Institute of Technology, Cambridge, Massachusetts, 02139, United States of America; 3 Institute for Medical Engineering and Science, Massachusetts Institute of Technology, Cambridge, Massachusetts, 02139, United States of America; 4 Harvard-MIT Division of Health Science and Technology, Massachusetts Institute of Technology, Cambridge, Massachusetts, 02139, United States of America; 5 Department of Anesthesiology, Boston Children’s Hospital, Boston, Massachusetts, 02115, United States of America; 6 Department of Microbiology and Immunobiology, Harvard Medical School, Boston, Massachusetts, 02115, United States of America; 7 Department of Molecular Medicine, Diabetes Center of Excellence, University of Massachusetts Medical School, Worcester, Massachusetts, 01605, United States of America; 8 Department of Pediatrics, University of Massachusetts Medical School, Worcester, Massachusetts, 01605, United States of America; 9 Department of Cancer Biology, University of Massachusetts Medical School, Worcester, Massachusetts, 01605, United States of America; 10 The Ragon Institute of MGH, MIT and Harvard, Cambridge, Massachusetts, 02139, United States of America; The Hospital for Sick Children and The University of Toronto, CANADA

## Abstract

*In vivo* implantation of sterile materials and devices results in a foreign body immune response leading to fibrosis of implanted material. Neutrophils, one of the first immune cells to be recruited to implantation sites, have been suggested to contribute to the establishment of the inflammatory microenvironment that initiates the fibrotic response. However, the precise numbers and roles of neutrophils in response to implanted devices remains unclear. Using a mouse model of peritoneal microcapsule implantation, we show 30–500 fold increased neutrophil presence in the peritoneal exudates in response to implants. We demonstrate that these neutrophils secrete increased amounts of a variety of inflammatory cytokines and chemokines. Further, we observe that they participate in the foreign body response through the formation of neutrophil extracellular traps (NETs) on implant surfaces. Our results provide new insight into neutrophil function during a foreign body response to peritoneal implants which has implications for the development of biologically compatible medical devices.

## Introduction

Biomaterials, drug delivery systems and medical devices are implanted into the body for a variety of therapeutic applications [1–4]. Often, foreign body responses against these implants result in the development of a fibrotic capsule that leads to their operational failure [5–8]. Foreign body responses begin with the deposition and denaturation of proteins on implant surfaces, followed by an inflammatory immune response. Numerous innate immune cells have been shown to participate in these responses, and potential roles for mast cells [9] as well as monocytes and macrophages [5–8,10] have been described. The precise role of neutrophils, another cellular component of the innate immune system, remains unclear.

Neutrophils are the first responders to both sites of invading pathogens and sterile inflammation caused by implantation of biomaterials. The primary function of neutrophils is the establishment of an acute inflammatory environment through degranulation, secretion of chemokines/cytokines, and phagocytosis of foreign substances [11–13]. These functions of neutrophils have been assessed, primarily, using either a microbial-infection [12,13] or chemical-induced inflammation model [14,15]. It remains to be determined, if these changes occur in neutrophils responding to sterile implant materials. Neutrophils have been shown to be present at implant sites during the acute stages of inflammation (2–3 days) [5,16,17] and have been suggested to have a high turnover rate [18]. Further, they have been shown to be involved in the degradation of implant materials through the release of oxidants [19–21]. However, evidence for their presence at implant sites beyond the early time-points (2–3 days) and their contribution to the inflammatory foreign-body response has been speculative.

Further, recent reports have described an additional role for neutrophils. In response to invading microbes, neutrophils have been shown to undergo an alternative cell death process that leads to the formation of granular protein and chromatin based neutrophil extracellular traps (NETs) [11,22,23]. Although the mechanism of their formation is not completely understood, they are known to be made of DNA and histone proteins, and also contain granular proteins such as neutrophil elastase [22]. NETs are believed to be a strategy employed by neutrophils to trap microbes *in vivo*, potentially as a response to infectious agents that are too large (generally larger than 10 μm in any one dimension) for neutrophil phagocytosis [24]. In light of these reports, we sought to also examine if such structures might be generated by neutrophils in response to large implants that cannot be taken up neutrophils through phagocytosis.

## Results

### Immune responses to peritoneal implants

**Fig 1:**
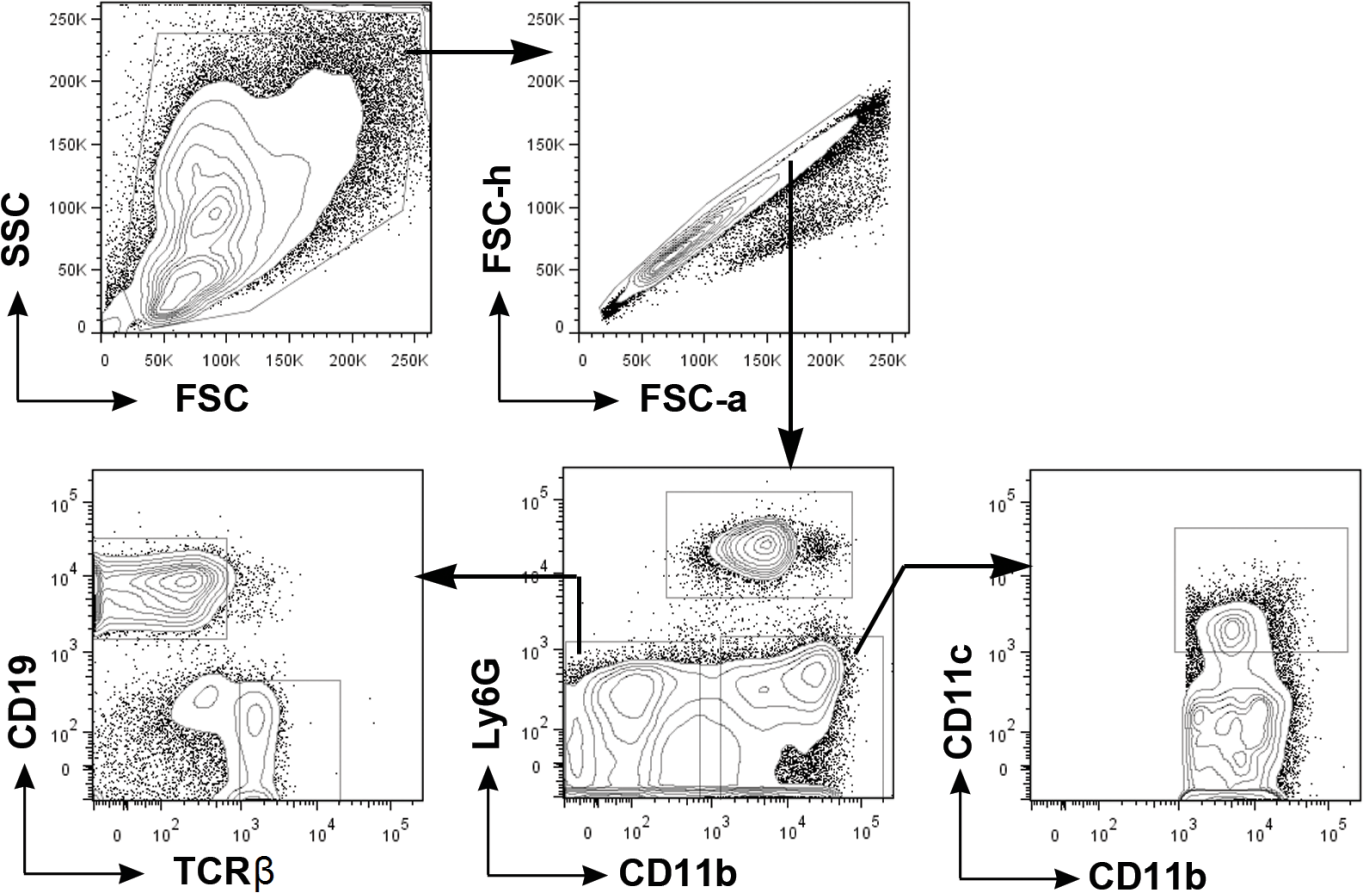
Flow cytometry schematics. Representative flow cytometry contour plots describing the gating scheme used to identify different immune cell subsets (isolated 2 weeks followed alginate microcapsule implantation) in the peritoneal cavity. All single cells retrieved from the peritoneal cavity were run through flow cytometry.

**Fig 2:**
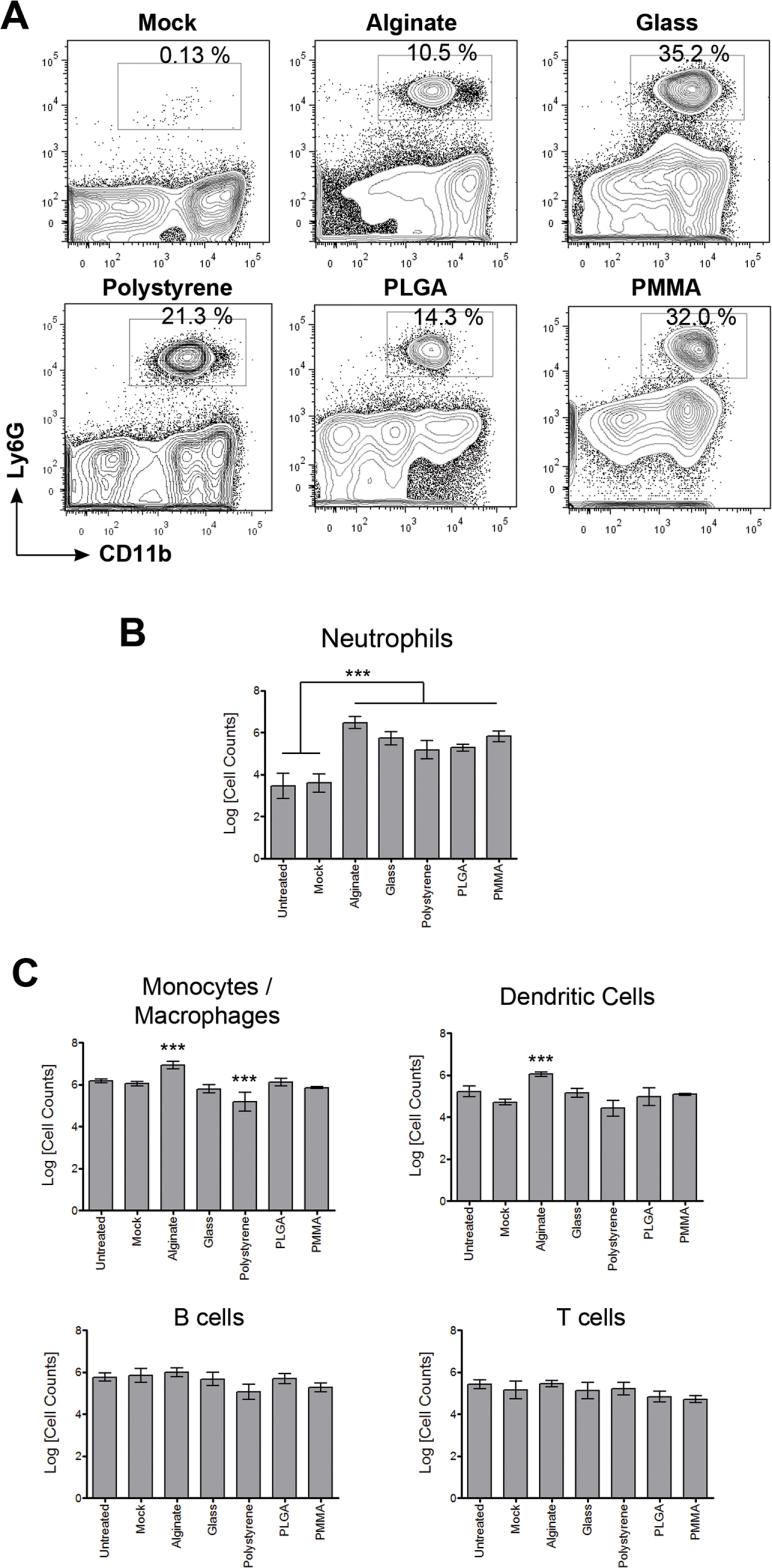
Increased neutrophil presence in peritoneal exudate following microcapsule implantation. (A)–Representative flow cytometry contour plots showing percentages of neutrophils (CD11b^+^ Ly6G^+^) in the peritoneal exudate of mice implanted with microcapsules made of different materials. (B)–Counts of neutrophils in the peritoneal exudate 2 weeks following implantation of microcapsules made of different materials compared to control untreated and mock treated mice. **C**–Counts of monocyte/macrophage (CD11b^+^ Ly6G^-^ CD11c^-^), dendritic cells (CD11b^+^ CD11c^+^), B cells (CD19^+^), and T cells (TCRβ^+^) in the peritoneal exudate 2 weeks following implantation of microcapsules made of different materials compared to control untreated and mock treated mice. Mock treatment entailed performing a laparotomy and injecting sterile saline (sham surgery). *** indicates p<0.001, using one-way ANOVA followed by Bonferroni post-test comparing specific sample to mock or untreated. Data are representative of at least 2 independent experiments with total n ≥ 5.

Using a mouse model of peritoneal implantation we characterized immune infiltrates in the peritoneal cavity, by flow cytometry (Fig 1), in response to implantation of microcapsules made of 5 different materials (Table 1). The combination of antibodies chosen to characterize cells in the peritoneal exudate were used to identify neutrophils, monocytes/macrophages, dendritic cells, B cells and T cells. Under homeostatic conditions, it has been shown that the peritoneal exudates contain resident macrophages, dendritic cells, B cells, and T cells [25]. Here we observe that following microcapsule implantation, a significant proportion (8–35%) of the peritoneal exudate is comprised of cells that expressed the cell-surface receptor Ly6G (Fig 2A), which have previously been identified as mouse neutrophils [26,27]. Increased neutrophil presence is expected at early time points (2–3 days) following implantation due to surgical trauma. But here we observe a 30–500 fold increase in neutrophil numbers 2 weeks following microcapsule implantation, compared to untreated or mock-transplanted mice receiving saline (Fig 2B). Changes in the numbers of other immune cells were minimal, with macrophages and dendritic cells numbers increasing by 5–10 fold only in the alginate microcapsule implanted mice (Fig 2C). Increases in neutrophil numbers was not limited to microcapsules that were spherical in shape, as similar increases were observed following implantation of non-spherical devices of 3 different shapes (S1 Fig).

**Table 1:** Sizing and counts of microcapsules used as models for device implantation in the peritoneal cavity of mice.

Microcapsules or other shaped devices	Size	Counts per 100 μl
Alginate (fabricated in the laboratory)	474.19 ± 21.7 μm in diameter; Intra-batch deviation = 55.4	1010 ± 160
Glass (acquired from Polysciences)	420–500 μm in diameter	1192 ± 161
Polystyrene (acquired from Polysciences and Phosphorex)	500–600 μm in diameter	N.D.
PLGA (acquired from Phosphorex)	512.1 ± 50.2 μm in diameter	N.D.
PMMA (acquired from Phosphorex)	497.3 ± 59.3 μm in diameter	N.D.
Alginate (~ 300 μm)	exact size not determined	2615 ± 304
Alginate (~ 800 μm)	exact size not determined	212 ± 28
Alginate (~ 2000 μm)	exact size not determined	9
PLGA–low molecular weight (fabricated in the laboratory)	256.66 ± 61.1 μm in diameter	N.D.
Alginate (threads)	~ 200 μm in diameter, > 10 cm in length	-
Alginate (cylinders)	~ 200 μm in diameter, 1–20 mm height	N.D.

N.D. = not determined. Mice were implanted with ~350 μl of microcapsules in all experiments. Total number of microcapsules implanted were calculated by multiplying the counts of microcapsules measured in 100 μl by a factor of 3.5, for data presented in supplementary Fig 3.

### Sterility and absence of endotoxin contaminants

**Fig 3:**
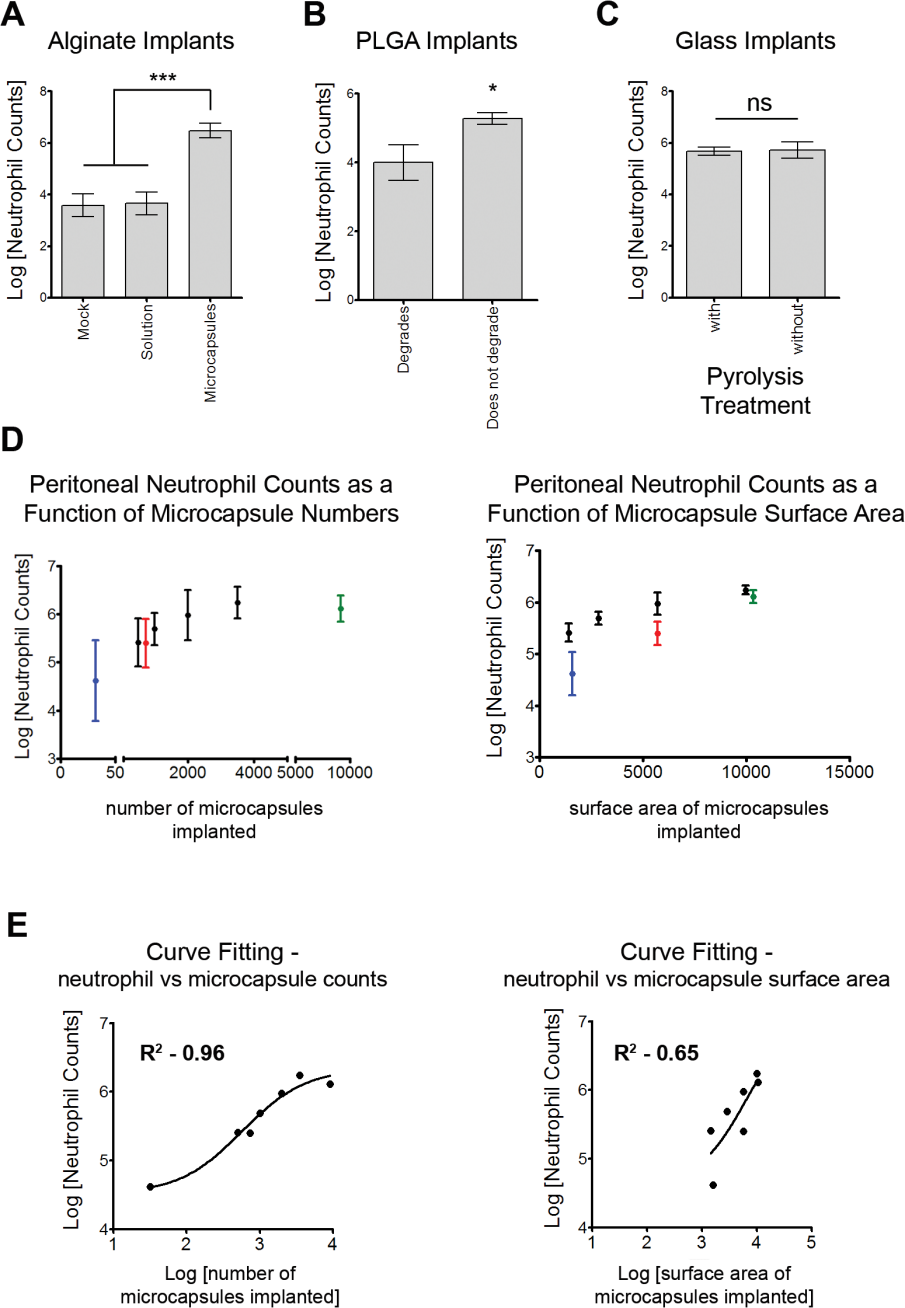
Increased neutrophil presence is due to biomaterial implants. (A)–Comparison of neutrophil counts in mice implanted with alginate in its solution or cross-linked hydrogel microcapsule form (measured 2 weeks following implantation). Mock and alginate microcapsule datasets are the same as presented in Fig 2. (B)–Comparison of neutrophil counts in mice implanted with PLGA that will or will not degrade in 2 weeks (measured 2 weeks following implantation). Datasets on PLGA microcapsules that do not degrade are the same as presented in Fig 2. Data are based on at least 2 independent experiments with n ≥ 5 for A and B. *** indicates p<0.001, using one-way ANOVA followed by Bonferroni post-test. (C)–Comparison of neutrophil counts in mice implanted with glass microcapsules that did or did not undergo pyrolysis treatment. Data are based on at least 1 independent experiment with n ≥ 4. 'ns' indicates not significant. Datasets on glass microcapsules that were not pyrolysis treated are the same as presented in Fig 2. (D)–Dependence of number of neutrophils observed in the peritoneal exduate on the number of microcapsules implanted, and less to absence of dependence on surface area of microcapsules. Black dots represent ~500μm, green dot represents ~300μm, red dot represents ~800μm and blue dot represents ~2000μm alginate microcapsules. Data are based on at least 1 independent experiment with n ≥ 5 mice for each microcapsule count. (E)–Curve fitting for data presented in 'D'. A non-linear 3-parameter dose response curve (mean of Log[neutrophil counts] vs Log[microcapsule count] or Log[microcapsule surface area]) was fitted assuming that microcapsules act as a stimulant for neutrophils. R^2^ value was 0.96 for the fit using the microcapsule counts, suggesting a direct correlation between neutrophil counts and microcapsule counts.

Increased neutrophil presence, above what is expected due to surgical trauma has generally been attributed to microbial contaminants (live/dead microbes as well as endotoxin). Importantly, both the implanted microcapsules and injected saline had undetectable endotoxin levels as determined using the limulus amebocyte lysate assay (S1 Table). In addition, the absence of an active infection in the peritoneal cavity was confirmed through endotoxin testing as well as swab cultures (for detection of live microbes) of the peritoneal fluid (S1 Table). Notably, neutrophil numbers increased in response to implantation of alginate when prepared as a cross-linked hydrogel microcapsule, but not when alginate was implanted as a solution (Fig 3A). Complementing this data, we observed significantly lower neutrophil levels 2 weeks following implantation of degradable PLGA microcapsules, when compared to PLGA microcapsules that do not degrade within this timeframe (Fig 3B). In addition, glass microcapsules that did or did not undergo pyrolysis treatment, for removal of endotoxins, prior to implantation in mice had similar increases in neutrophil numbers (Fig 3C). Also, the increase in neutrophil numbers were directly dependent on the number of microcapsules implanted, and do not appear to be dependent on surface area of implanted material (Fig 3D and 3E). Further, minimal changes in the weight of animals (S2 Fig) suggests the absence of an infection.

### Neutrophil Function—phagocytosis and inflammatory cytokine/chemokine secretion

**Fig 4:**
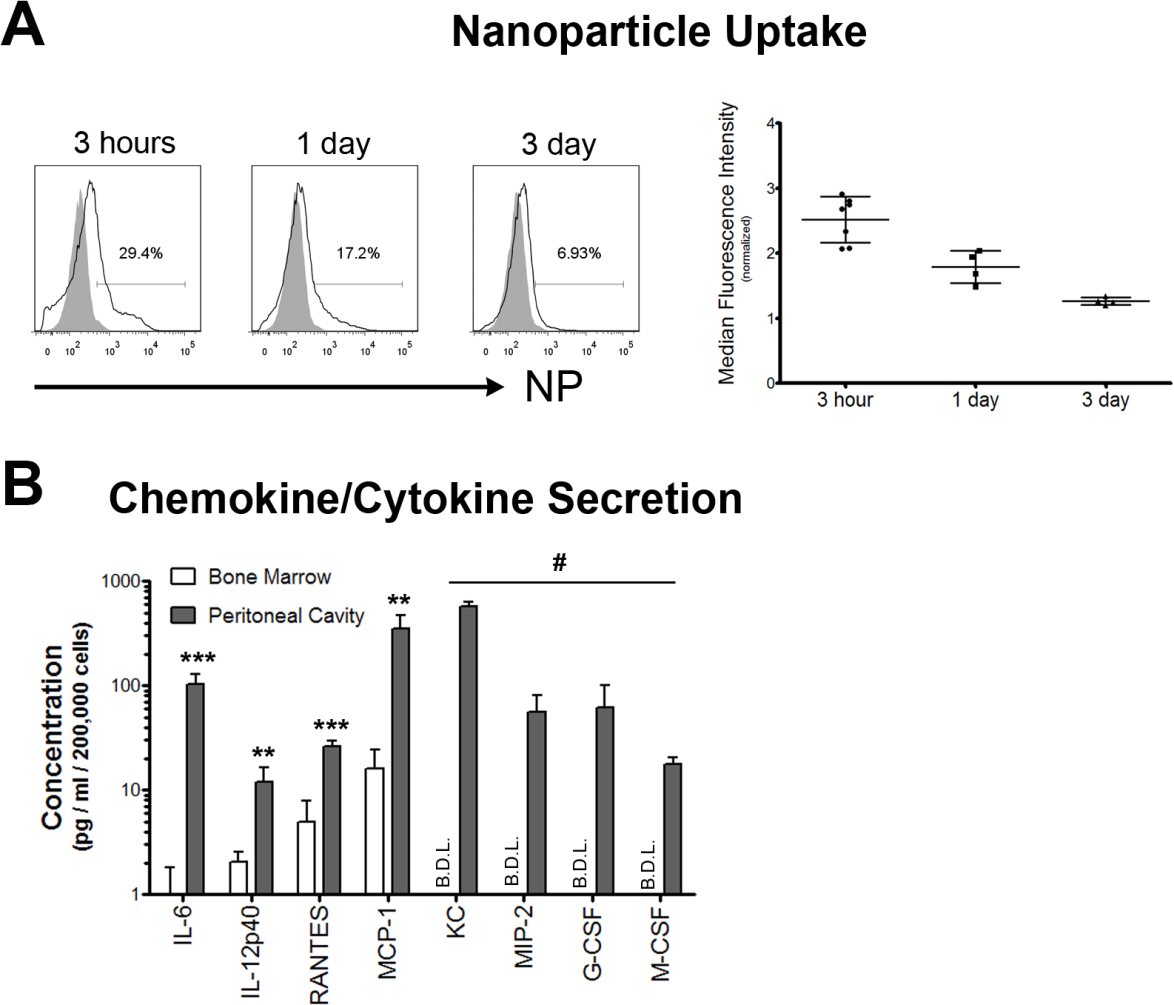
Neutrophil Function. (A)–Confirmation of neutrophil phagocytic capacity. Fluorescent nanoparticles (~190 nm polystyrene nanoparticles) were injected intraperitoneally, 1 week following alginate microcapsule implantation. *Left*–Representative flow cytometry histograms generated following gating on Ly6G^+^ cells showing nanoparticles (NP) associated with neutrophils. Grey histograms are fluorescence intensities in control mice that have not been injected with nanoparticles. *Right*–Quantification of the NP uptake histograms, showing a large increase in NP MFI 3 hours post NP injection that drops over time. Data are representative of at least 1 independent experiment with total n ≥ 4. (B)–Multiplex luminex assay to measure chemokines and cytokines secreted by neutrophils. Neutrophils were isolated using a magnetic bead based negative selection technique, followed by *ex vivo* overnight culture. Higher amounts of key inflammatory cytokines and chemokines are secreted by peritoneal cavity but not bone marrow neutrophils. B.D.L. = below detectable levels. ** and *** indicate p<0.01 and p<0.001, respectively, using a two-tailed Student's t test with Welch's correction (for samples where the levels of cytokine/chemokine are above detectable levels). # indicates p<0.01 using a two-tailed Fisher's exact test, for samples where the levels of cytokine/chemokine were below detectable levels. Data presented are based on n = 6.

In response to biomaterial or device implantation, neutrophils have been reported to phagocytose the implanted material if it is small enough, and/or establish an inflammatory environment through the secretion of cytokines and chemokines. The microcapsules implanted in the peritoneal cavity were too big to be taken up by neutrophils (all diameters >250 μm). Hence, to evaluate phagocytic capacity of neutrophils present in the peritoneal cavity, fluorescently tagged polystyrene nanoparticles were administered following implantation of microcapsules. A significant percentage of peritoneal neutrophils were associated with fluorescent nanoparticles, confirming their phagocytic capacity (Fig 4A). To test for cytokine/chemokine secretion capacity, neutrophils from the peritoneal cavity of alginate microcapsule implanted mice were purified using magnetic bead-based purification and cultured overnight ex vivo (~200,000 per well). Neutrophils from mock control animals were not tested in this assay due to the very low numbers of these cells (<5000 per mouse). Instead, bone marrow neutrophils were used as controls in this assay, as these cells are thought to be naïve (not activated and secreting inflammatory mediators) [28,29]. The cytokines/chemokines present in the cell culture supernatant were then quantified using a multiplex luminex assay. Peritoneal-neutrophils secreted a number of cytokines and chemokines in significantly greater quantities (Fig 4B), when compared to bone marrow-neutrophils, which indicate their ability to promote the establishment of an inflammatory microenvironment and demonstrate that these cells are activated.

### Neutrophil Extracellular Traps

**Fig 5:**
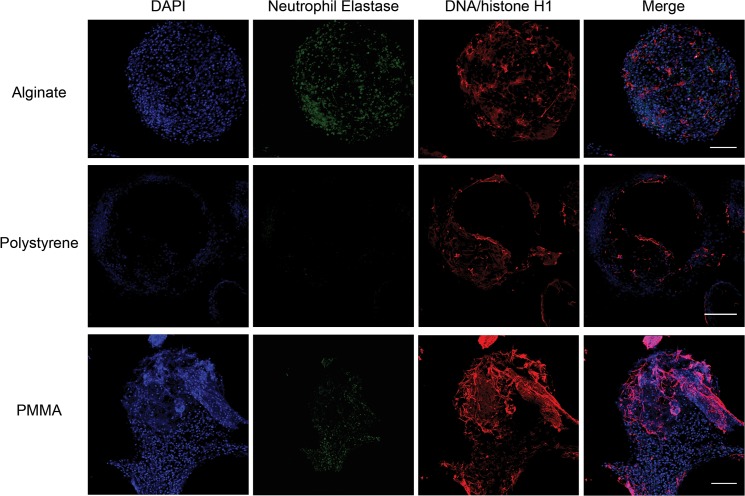
Neutrophil extracellular traps. Representative z-stacked immunofluorescence images showing neutrophil elastase and DNA/histone-H1 on the surface of microcapsules. Alginate microcapsules were retrieved 1–2 weeks following implantation, while Polystyrene and PMMA microcapsules were retrieved 3 days following implantation. Images are representative of at least 2 independent experiments with total n ≥ 5 mice, and imaging of multiple retrieved microcapsules from each mouse. Scale bar = 100 μm.

Next, we evaluated whether NETs were produced in response to implantation of devices, as the microcapsules we implant in the peritoneal cavity are too large to be taken up by neutrophils. Three days following implantation of either polystyrene or poly (methyl-methacrylate) microcapsules, extracellular deposits were observed on the microcapsule surface using scanning electron microscopy (S3 Fig). Immunofluorescent detection of DNA/Histone H1 and neutrophil elastase on polystyrene and PMMA microcapsule surfaces (3 days following implantation) as well as on alginate microcapsule surfaces (1 week following implantation), suggested that at least a few of these structures were neutrophil extracellular traps (Fig 5 and S4 Fig). Myeloperoxidase (Fig A in S5 Fig) and cirtullinated histone H3 (Fig B in S5 Fig) along with DNA (sytox based detection) were also observed, providing further support for NET formation on microcapsule surfaces. Additionally, a ~3-fold increase in neutrophil elastase was observed in the peritoneal exudate of mice implanted with alginate microcapsule when compared to saline controls (Fig C in S5 Fig).

## Discussion

Neutrophils are thought to have a limited role in responses to sterile inflammatory insults, such as implantation of a medical device [4–6,10,30,31], due to their relatively short life-span [32–34] and their primary role of killing microbes through degranulation [11–13]. However, the details of this limited role remain unclear. Neutrophils have been suggested to be present during the acute stages of inflammation [17], aid in implant degradation [19–21], and it has been speculated that they are involved in secreting inflammatory mediators at implant sites [5]. Here we present evidence that suggests neutrophils are present at peritoneal implant sites for a longer duration, demonstrate that they secrete a variety of inflammatory cytokines and chemokines, and describe a potential additional role for neutrophils at the biological-implant interface.

In a mouse model of peritoneal implantation using spherical microcapsules made of 5 different materials and implants made of 4 different shapes, we observe an increased neutrophil presence in the peritoneal exudates (Fig 2). Neutrophil presence beyond acute stages of surgical trauma (2–3 days following surgery) has been suggested to be a result of microbial contaminants [4,31]. Our observations of undetectable endotoxin levels (i) in the peritoneal fluid of implanted mice; (ii) on the devices themselves; (iii) in the materials used to make devices; and (iv) in the saline vehicle, support the absence of endotoxin in these studies (S1 Table). Additionally, all mice implanted with microcapsules remained healthy, as determined through measuring body-weight changes (S2 Fig) and with regular veterinary check-ups, suggesting the absence of an active infection associated with surgical implantation. We also observed that implantation of one of the materials (alginate) as a solution, when compared to its hydrogel state as a microcapsule device, did not result in increased neutrophil presence (Fig 3A). Further, we show that microcapsules that degrade do not leave a residual neutrophil presence (Fig 3B). Put together, these data strongly suggest that the neutrophil response observed in this model of peritoneal implantation is not due to a contaminating microbial inflammatory stimulus (either endotoxin or active microbial infection), but rather due to the presence of foreign device materials in the peritoneal space.

Neutrophils function primarily through phagocytosis and the secretion of inflammatory cytokines/chemokines. We confirm the phagocytic capacity of neutrophils through their ability to take up fluorescent nanoparticles co-injected with larger implants (Fig 4A). Neutrophils recruited to the peritoneal cavity in response to chemical stimuli have enhanced expression of a variety of inflammatory chemokines/cytokine genes, when compared to their "non-inflammatory" bone marrow counterparts [29]. We sought to determine if such changes would be observed in neutrophils recruited to the peritoneal cavity in response to implant materials. Magnetic bead based neutrophil purification followed by their overnight ex vivo culture, confirms the ability of peritoneal neutrophils to secrete increased amounts of inflammatory mediators, when compared to bone marrow neutrophils, which alludes to their activated state (Fig 4B).

To determine if neutrophils form extracellular traps as a component of their response to implanted devices, we performed multiple imaging studies on the surface of implants. Scanning electron micrographs of implants of two different materials (polystyrene and PMMA) showed extracellular deposits on the surface (S3 Fig). Although, these structures could be cellular extensions and/or protein deposits, the presence of a few long, thin (<50nm width) fibers suggested that at least some of these structures could be extracellular DNA/chromatin fibers. Detection of DNA/Histone-H1 and neutrophil elastase (Fig 5), as well as the combination of DNA (Sytox), myeloperoxidase, citrullinated histone H3 and histone-H1 (Figs A and B in S5 Fig) [22,23] supports NET formation on the implant surface. Overall, these data indicate that neutrophils more closely interact with implanted foreign bodies and potentially contribute to deposition of NETs on their surface.

The peritoneal model for implantation was chosen for the ease in counting and characterizing (using flow cytometry) immune infiltrates [35–37]. It remains to be seen if short-term (up to 2 weeks) increases in neutrophil presence, in the absence of microbial contaminants, would be observed in other tissues as well, given multiple established reports suggesting that neutrophil presence is limited at subcutaneous and intramuscular implant sites (reviewed in [5,6]). Importantly, we observe at least a few NET-like structures on a majority of alginate microcapsules at 1 week post implantation, and PMMA and polystyrene microcapsules at 3 days post implantation (Fig 5). However, currently, we are unable to quantify NET formation on individual microcapsules or the implant as a whole. Additionally, the kinetics as well as the factor/s and signaling cascades eliciting NET formation on implant surfaces remain unclear. Further research is required to determine the role of NETs in the overall fibrotic reaction and if NETs on implant surfaces could act to nucleate implant fibrosis.

## Conclusion

In conclusion, we demonstrate significantly increased neutrophil presence in the peritoneal space 2 weeks following microcapsule implantation. These increases are due to sterile inflammatory responses against the implant and not due to endotoxin or microbial contamination. The recruited neutrophils are activated, secrete inflammatory mediators and potentially form NETs on implant surfaces. These results suggest an additional role for neutrophils in the foreign body response against implanted devices, which could have potential implications in developing more compatible medical devices and providing new drug targets to prevent implant fibrosis. Further, this peritoneal microcapsule implantation model could help in elucidating the broader roles of neutrophils in sterile inflammatory reactions.

## Materials and Methods

### Mice

Male C57BL/6J mice were purchased from Jackson Laboratories (Bar Harbor, Maine, USA). Mice in the age range of 8–12 week (corresponding to 20–25 grams for male were used in all experiments. All animal experiments described in this study were specifically approved by the Massachusetts Institute of Technology and Boston Children's Hospital Committee on Animal Care, and followed federal and state regulations. Mice were euthanized using carbon dioxide asphyxiation followed by cervical dislocation as a secondary method, as approved by the MIT committee on animal care

### Alginate microcapsules

Alginate microcapsules were prepared using an electrostatic droplet generator as described [38]. Briefly, SLG20 alginate (Nova Matrix, FMC BioPolymer, Drammen, Norway) was dissolved in 0.9% (w/v) NaCl solution to a concentration of 1.4% (w/v). For 500 μm sized microcapsules, alginate solution was passed through a 25G blunt needle, at a flow rate of 0.18 ml/min and a voltage of approximately 5 kV, into a 20 mM BaCl_2_ cross-linking solution. For 300 μm, 800 μm and 2000 μm sized capsules, alginate was passed through a 27G, 25G and 18G blunt needle, respectively. Capsules were collected and washed six times in HEPES buffer (132 mM NaCl, 4.7 mL KCl, 25 mM HEPES, 1.2 mM MgCl_2_) and stored overnight at 4C in HEPES buffer. Prior to *in vivo* implantation, the alginate microcapsules were washed six times in 0.9% (w/v) NaCl solution. All materials used in the preparation of these microcapsules were endotoxin free (endotoxin below detection limits as stated by manufacturers). Alginate microcapsules were prepared inside a biological laminar flow hood and all solutions used (saline, HEPES, BaCl_2_) were autoclaved prior to use.

### Glass, polystyrene and PMMA microcapsules

Glass microcapsules were purchased from Polysciences Incorporated (Warrington, PA, USA), polystyrene microcapsules from Polysciences or Phosphorex (Hopkinton, MA, USA), and poly methyl-methacrylate microcapsules (PMMA) were purchased from Phosphorex. Microcapsules were washed with 70% ethanol (4 times), transferred to a biological laminar flow hood, and washed with sterile saline (4 times) prior to implantation in mice. All microcapsules were handled in endotoxin, DNase and RNase free microcentrifuge tubes.

### PLGA microcapsules

Poly (lactic-co-glycolic) acid (PLGA) microcapsules that do not degrade in 2 weeks (made of polymer with inherent viscosity of 0.4–0.5) were purchased from Phosphorex. While PLGA microcapsules that degrade in 2 weeks (made of polymer with inherent viscosity of 0.16–0.25) were prepared using a single emulsion-evaporation technique in our laboratory. Degradation of these capsules was tested by incubating in saline at 37°C in vitro (with regular changes in saline) as well as by *in vivo* implantation (no capsules visible for retrieval 2 weeks post implantation).

In most experiments (unless specified otherwise), microcapsules with a diameter of ~500 μm were used and approximately 3500 microcapsules were implanted per mouse.

### Pyrolysis treatment

Pyrolysis of glass microcapsules was performed by alternately heating microcapsules placed in a glass container to 240°C for 1 hour and sonicating for 20 min (repeated 3 times). Microcapsules that underwent pyrolysis were transferred to a biological laminar flow hood, washed with sterile saline and implanted in mice.

### *In vivo* surgical implantation of microcapsules

Mice were anesthetized using isoflurane in oxygen at a flow rate of 2.5 L/min. A 1 cm incision was made on the skin surrounding the abdomen and the microcapsules were either injected through the peritoneal wall using an 18 gauge needle or implanted using a sterile transfer pipet following a 0.5–1 cm incision along the midline in the peritoneal wall. The incision in the peritoneal wall was closed using a 6–0 taper polydioxanone (PDS II) suture. The skin incision was closed using a reflex wound clip system and VetBond tissue adhesive. All animals received subcutaneous buprenorphine (0.05 mg/kg) preoperatively and every 8–12 hours for 2 days postoperatively. Additionally, subcutaneous saline was provided to animals as required.

Microcapsules (approximately 350 μl) were suspended in an additional 0.5 ml of 0.9% NaCl for implantation. The entire volume of ~850 μl was injected into the peritoneal cavity during surgery. In mock controls (also called sham surgery), the animals underwent surgical procedures as described above with one modification–the injected material was ~850 μl of saline (no microcapsules). In alginate solution injected animals, an un-cross-linked solution of SLG20 alginate (350 μl) was suspended in an additional 0.5 ml of 0.9% NaCl prior to injection.

### Retrieval of cells and implants

For retrievals, mice were euthanized through gaseous CO_2_ administration followed by cervical dislocation. Immediately following euthanasia, 5 ml of cold phosphate buffered saline (PBS) was injected into the peritoneal cavity using a 25G needle. Through a small incision in the peritoneal wall, cells and peritoneal fluid were retrieved, passed through a 70 μm filter (to filter out implants) and stored on ice prior to analysis. Microcapsules were collected on the 70 μm filter by rinsing the peritoneal cavity with Krebs-Henseleit solution and stored in this solution or fixative (2% paraformaldehyde) prior to analysis.

### Cell counts

All cells isolated from the peritoneal exudate were subjected to red blood cell lysis, following which single cell suspensions were prepared. Total live cell counts (Trypan Blue negative) were determined by using the automated cell counter, Countess (Life Technologies, Grand Island, NY, USA). To determine, total counts of neutrophils, the total live cell counts were multiplied by the percentage of cells that were Ly6G^+^ CD11b^+^ of the singlet population on flow cytometry.

### Flow cytometry

Single cell suspensions from the peritoneal cavity were prepared in PBS containing 0.5% bovine serum albumin and 2 mM EDTA (staining solution). Cells were stained with the following antibodies to surface receptors (table below) for 20 minutes at 4C in the presence of Fc Block prior to data collection on a BD-LSR II, BD-LSR Fortessa or BD-LSR Canto. Data were analyzed using FlowJo (Tree Star Inc., Ashland, OR, USA). The following antibodies were used: Ly6G (clone 1A8), Ly6C (clone HK1.4), CD11b (clone M1/70), CD19 (clone 6D5), TCRβ (clone H57-97), CD11c (clone N418), F4/80 (clone BM8), CD115 (clone AFS98), and CD14 (clone Sa14-2). All antibodies were purchased from Biolegend (San Diego, CA) or eBioscience (San Diego, CA).

### Imaging of microcapsules

Retrieved microcapsules were washed with Krebs-Henseleit solution prior to phase contrast imaging, or were fixed in 2% paraformaldehyde (in PBS) for 4 hours. Following multiple washing steps (washing buffer–PBS containing 1% BSA), microcapsules were suspended in staining solution (same as in flow cytometry) in 1.5 ml micro-centrifuge tubes. Primary antibodies against, Ly6G-Alexa-Fluor-647 (1A8, Biolegend; 1:50 dilution), DNA/Histone H1 (MAB3864, Millipore; 1:200 dilution), neutrophil elastase (ab21595, Abcam; 1:200 dilution), Histone-H1 (ab61177, Abcam; 1:250 dilution), citrulline Histone-H3 (ab5103, Abcam; 1:100 dilution), and/or myeloperoxidase (ab90810, Abcam; 1:50 dilution) were added and left on a gentle rocker for 1 hour at 4°C. Following multiple washes, secondary antibodies were added at 1:1000 dilution and left on a gentle rocker for 1 hour at 4C. Again, after multiple washes, microcapsules were suspended in PBS and 500nM DAPI and/or Sytox red (Life Technologies) added for 15 min at room temperature. Following 3 washes using PBS, microcapsules were suspended in 50:50 glycerol:PBS solution and saved at 4°C for imaging. None of the steps involved addition of tween or other detergents for cell permeabilization. Immunofluorescence imaging was performed using a Zeiss LSM-700. Laser power and gain settings were adjusted using control samples that were stained with secondary antibodies only, and kept constant while imaging all the samples. Z-stacks of individual capsules (50–100 μm depth) were collected with a 4–5 μm section interval, and a 3-D stack (represented as a 2-D image) was generated using 3D reviewer on the Zeiss Zen software. Three post image acquisition processing steps were performed on FIJI: (i) adjustment of brightness and contrast to the entire image (including control samples), (ii) cropping images to limit the region of interest to individual capsules and (iii) the addition of a scale-bar.

### Neutrophil isolation

Neutrophils were isolated from a mixed population of peritoneal cavity and bone marrow cells using a magnetic bead based negative selection kit (Stemcell Technologies, Vancouver, BC, Canada) in accordance with the manufacturer's protocol with one modification: in addition to the antibodies provided by manufacturer, we added purified anti-mouse CD115 (clone AFS98) at 1:250 dilution to the cells. Following incubation with the antibodies, magnetic beads for the separation of antibody bound cells were added. Bead bound cells were removed from the cell mixture, leaving behind a purified population of neutrophils.

### Multiplex Luminex assay

Purified neutrophils were cultured overnight in cell culture media (DMEM–low glucose content from Life Technologies containing 10% fetal bovine serum and 1% antibiotics). The control for this assay was media left overnight in the same plate without cells. Following overnight culture, cell culture supernatant was collected and stored at -80C prior to assay. A BioRad (Hercules, CA, USA) 32-plex mouse cytokine, chemokine assay was used according to manufacturer's instructions. Data was collected using a Bio-Rad 200 system.

### Neutrophil elastase measurement

To collect peritoneal fluid the peritoneal cavity of euthanized mice was first injected with 2.5 ml of sterile PBS. Peritoneal fluid along with PBS was retrieved and passed through a 70 μm filter to remove implanted microcapsules. Under sterile conditions, the fluid was passed through a 5 μm syringe filter prior to storage at -80C. A separate cohort of mice were used for these experiments. Neutrophil elastase activity in this solution was measured using a kit purchased from Cayman Chemical Company (Ann Arbor, MI, USA) and used according to manufacturer's instructions. Neutrophil elastase activity data from mock and microcapsule implanted animals was normalized to untreated controls.

### Nanoparticle uptake experiments

Mice were implanted with alginate microcapsules to elicit neutrophil recruitment. One week following implantation, fluorescent polystyrene nanoparticles (NP)– 200 μl of Flash Red 190nm PS particles (Bangs Laboratories Inc., Fishers, IN, USA) per mouse, were injected intraperitoneally. At different time points following NP injection peritoneal exudates were recovered and NP associated with Ly6G^+^ cells were determined using flow cytometry.

### Statistics

All data presented in the main paper are based on at least 2 or more independent experiments with a total of at least 5 animals per experimental group. An independent experiment is described as an experiment involving new/different batches of microcapsules, reagents, mice and performed on a separate date. Each 'n' represents an individual animal (for *in vivo* studies) or samples pooled together from one or multiple animals (for ex vivo studies). All data were analyzed and graphs generated using GraphPad Prism 5 (GraphPad Software, La Jolla, CA, USA). One-way ANOVA was used for all statistical comparisons involving multiple groups, and an unpaired Student's t-test with Welch's correction was used for comparisons between 2 groups. For the multiplex luminex assay samples that were below detectable levels (hence no numerical value associated with them), a two-tailed Fisher's exact test was used for statistical analysis, in which the data was represented as categories (detectable vs. non-detectable). Significance is represented as * p<0.05, ** p<0.01, *** p<0.001 and # p<0.01. Data are always presented as mean ± standard deviation.

## Supporting Information

S1 Fig Effect of implant shape on neutrophil numbers. Neutrophil presence in response to alginate implants that were spheres (*same as data presented in Fig 2*), threads, cylinders or irregular-shaped was determined. Data are based on at least 1 independent experiment with n ≥ 3 mice.
(PDF)

S2 Fig Weight changes in mice. No significant changes were observed in the weight of mice that were mock treated or implanted with alginate microcapsules. An expected drop in weight was observed following surgery (in both mock and microcapsule implanted), but the weights quickly recovered and by 2 weeks following surgery the mice had started to gain weight.
(PDF)

S3 Fig Neutrophil extracellular traps (SEM). Scanning electron micrographs of polystyrene and PMMA microcapsules retrieved 3 days following implantation in male C57BL/6J mice. Long, thin fibers are observed on the surface of the microcapsules that could potentially be part of neutrophil extracellular traps. Scale bars on images are 5 μm, except for PMMA extreme right (1 μm) image. Images are representative of 2 independent experiments with n ≥ 5.
(PDF)

S4 Fig Addendum to Fig 5 in manuscript. Additional images of data presented in Fig 5. See legend of Fig 5 for details.
(PDF)

S5 Fig Neutrophil extracellular traps (additional stains). (A)–Representative z-stacked immunofluorescence images showing histone-H1, myeloperoxidase (MPO) and DNA (sytox and DAPI) on the surface of microcapsules. Polystyrene and PMMA microcapsules were explanted 3 days following implantation. Scale bar = 100 μm. In merged images, blue represents sytox, red–MPO and green–Histone-H1. (B)–Representative z-stacked immunofluorescence images showing citrullinated histone-H3 and DNA (sytox and DAPI) on the surface of microcapsules. PMMA microcapsules were explanted 3 days following implantation. Scale bar = 100 μm. In merged image, blue represents DAPI, green–cit-histone-H3 and purple–sytox. Data in 'B' and 'C' are representative of 1 independent experiment with n = 3 mice, and imaging of multiple microcapsules explanted from each mouse. (C)–Measurement of neutrophil elastase activity in the peritoneal fluid of mock or alginate microcapsule implanted mice (2 weeks post implantation). * indicates p<0.05. Data are representative of 2 independent experiments with n = 6.
(PDF)

S1 Table Absence of microbial contaminants and infections in the peritoneal cavity. Endotoxin testing, culture of peritoneal fluid and general health of animals implanted with microcapsules suggested an absence of microbial contaminants or infections.
(PDF)
