# Supplementary material for: Neutrophil Responses to Sterile Implant Materials
Source: PLoS One. 2015 Sep 10;10(9):e0137550. doi: 10.1371/journal.pone.0137550 (PMC4565661; doi:10.1371/journal.pone.0137550)
Supplement: S1 Fig — Neutrophil presence in response to alginate implants that were spheres (same as data presented in Fig 2), threads, cylinders or irregular-shaped was determined. Data are based on at least 1 independent experiment with n ≥ 3 mice. (PDF) [file pone.0137550.s001.pdf]

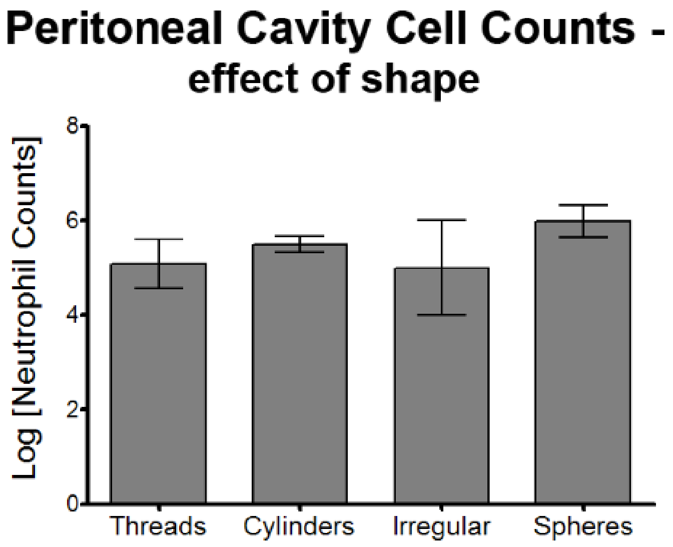

S1 Figure: Effect of implant shape on neutrophil numbers. Neutrophil presence in response to alginate implants that were spheres (*same as data presented in Fig. 2*), threads, cylinders or irregular-shaped was determined. Data are based on at least 1 independent experiment with n ≥ 3 mice
