# Supplementary material for: Neutrophil Responses to Sterile Implant Materials
Source: PLoS One. 2015 Sep 10;10(9):e0137550. doi: 10.1371/journal.pone.0137550 (PMC4565661; doi:10.1371/journal.pone.0137550)
Supplement: S2 Fig — No significant changes were observed in the weight of mice that were mock treated or implanted with alginate microcapsules. An expected drop in weight was observed following surgery (in both mock and microcapsule implanted), but the weights quickly recovered and by 2 weeks following surgery the mice had started to gain weight. (PDF) [file pone.0137550.s002.pdf]

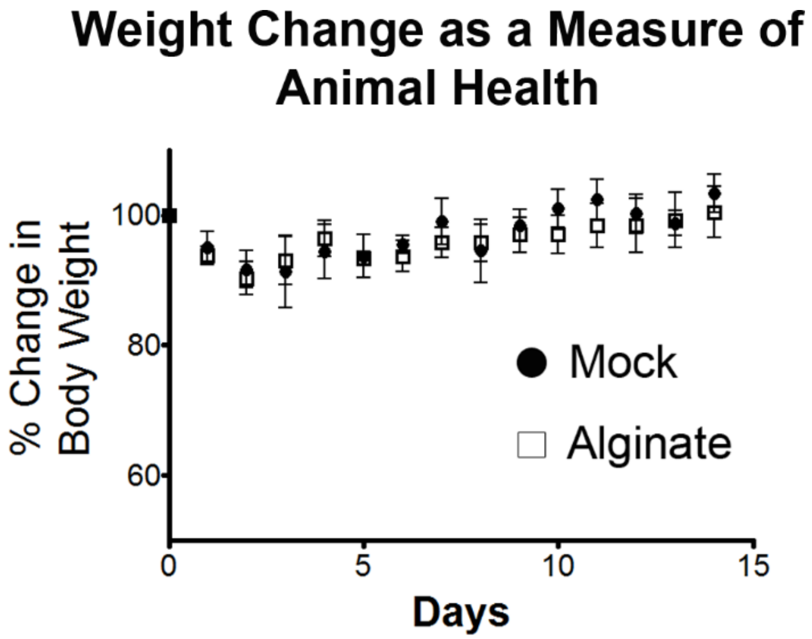


**S2 Figure: Weight changes in mice.** No significant changes were observed in the weight of mice that were mock treated or implanted with alginate microcapsules. An expected drop in weight was observed following surgery (in both mock and microcapsule implanted), but the weights quickly recovered and by 2 weeks following surgery the mice had started to gain weight.
