# Supplementary material for: Neutrophil Responses to Sterile Implant Materials
Source: PLoS One. 2015 Sep 10;10(9):e0137550. doi: 10.1371/journal.pone.0137550 (PMC4565661; doi:10.1371/journal.pone.0137550)
Supplement: S3 Fig — Scanning electron micrographs of polystyrene and PMMA microcapsules retrieved 3 days following implantation in male C57BL/6J mice. Long, thin fibers are observed on the surface of the microcapsules that could potentially be part of neutrophil extracellular traps. Scale bars on images are 5 μm, except for PMMA extreme right (1 μm) image. Images are representative of 2 independent experiments with n ≥ 5. (PDF) [file pone.0137550.s003.pdf]

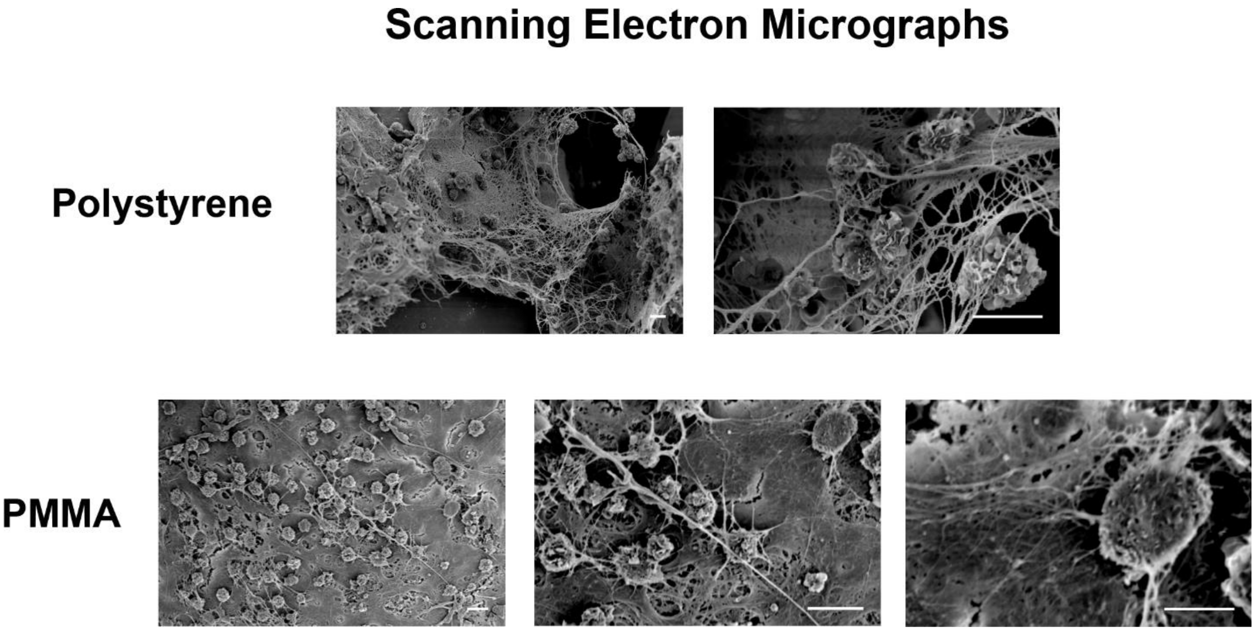

S3 Figure: Neutrophil extracellular traps (SEM). Scanning electron micrographs of polystyrene and PMMA microcapsules retrieved 3 days following implantation in male C57BL/6J mice. Long, thin fibers are observed on the surface of the microcapsules that could potentially be part of neutrophil extracellular traps. Scale bars on images are 5 µm, except for PMMA extreme right (1 µm) image. Images are representative of 2 independent experiments with n ≥ 5.
