# Supplementary material for: Neutrophil Responses to Sterile Implant Materials
Source: PLoS One. 2015 Sep 10;10(9):e0137550. doi: 10.1371/journal.pone.0137550 (PMC4565661; doi:10.1371/journal.pone.0137550)
Supplement: S4 Fig — Additional images of data presented in Fig 5. See legend of Fig 5 for details. (PDF) [file pone.0137550.s004.pdf]

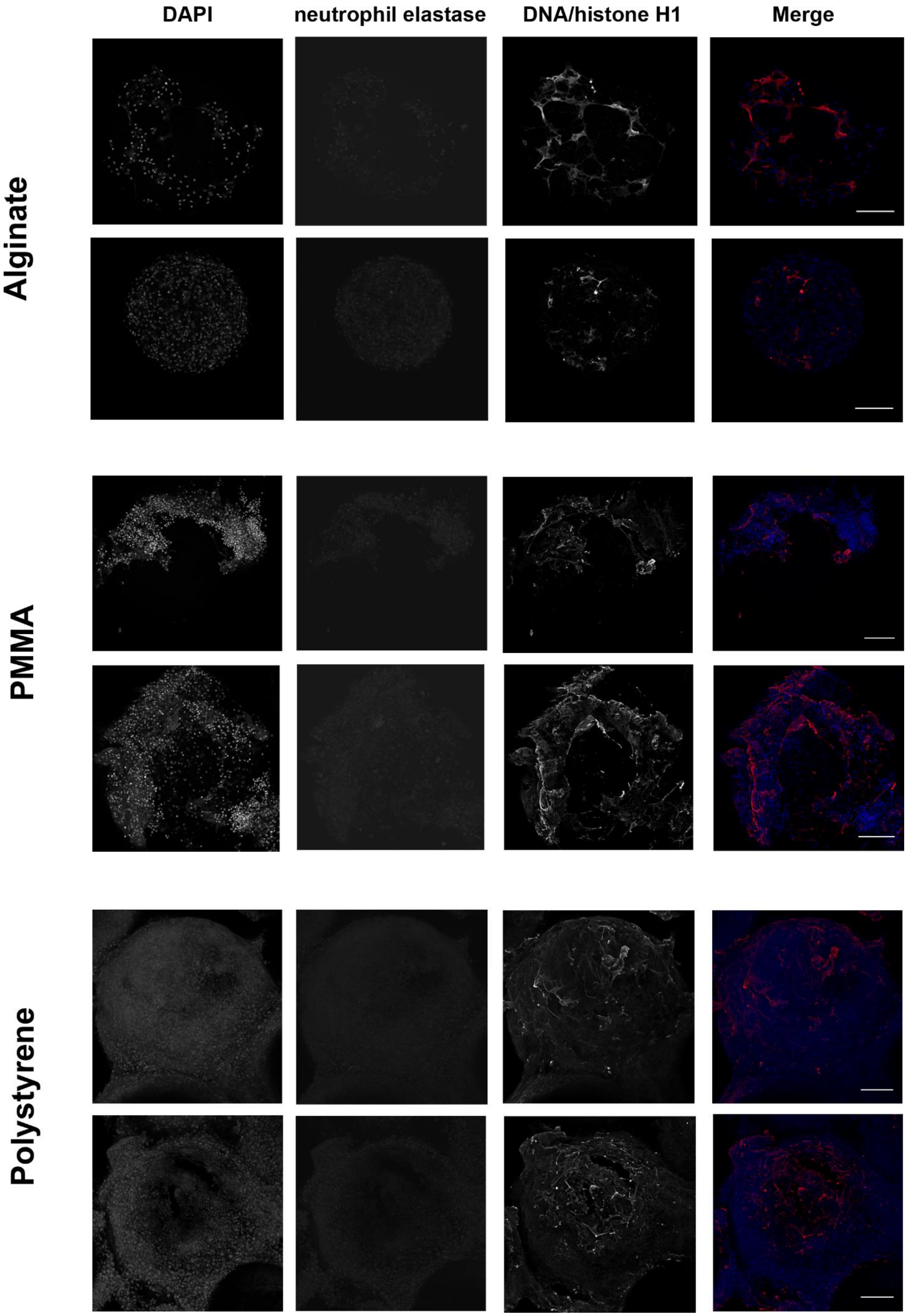

S4 Figure: Addendum to figure 5 in manuscript. Additional images of data presented in figure 5. See legend of figure 5 for details.
