# Supplementary material for: Neutrophil Responses to Sterile Implant Materials
Source: PLoS One. 2015 Sep 10;10(9):e0137550. doi: 10.1371/journal.pone.0137550 (PMC4565661; doi:10.1371/journal.pone.0137550)
Supplement: S5 Fig — (A)–Representative z-stacked immunofluorescence images showing histone-H1, myeloperoxidase (MPO) and DNA (sytox and DAPI) on the surface of microcapsules. Polystyrene and PMMA microcapsules were explanted 3 days following implantation. Scale bar = 100 μm. In merged images, blue represents sytox, red–MPO and green–Histone-H1. (B)–Representative z-stacked immunofluorescence images showing citrullinated histone-H3 and DNA (sytox and DAPI) on the surface of microcapsules. PMMA microcapsules were explanted 3 days following implantation. Scale bar = 100 μm. In merged image, blue represents DAPI, green–cit-histone-H3 and purple–sytox. Data in 'B' and 'C' are representative of 1 independent experiment with n = 3 mice, and imaging of multiple microcapsules explanted from each mouse. (C)–Measurement of neutrophil elastase activity in the peritoneal fluid of mock or alginate microcapsule implanted mice (2 weeks post implantation). * indicates p<0.05. Data are representative of 2 independent experiments with n = 6. (PDF) [file pone.0137550.s005.pdf]

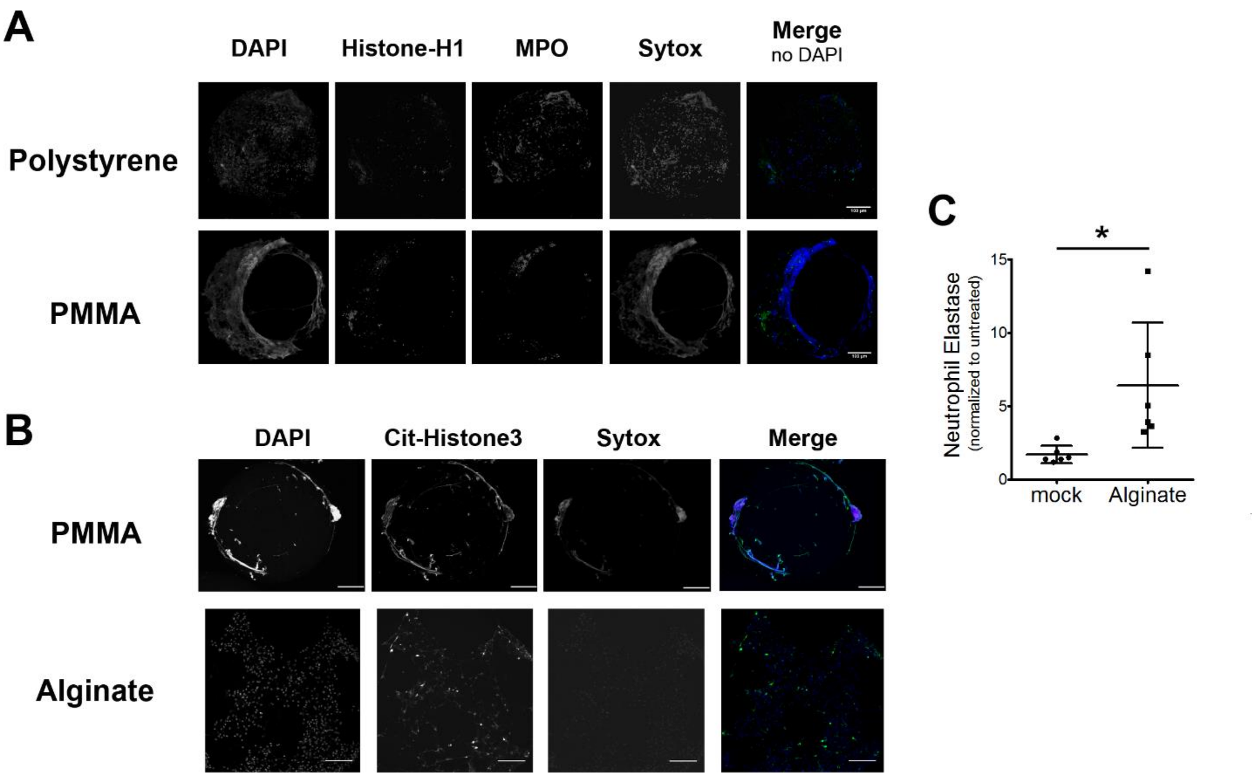

S5 Figure: Neutrophil extracellular traps (additional stains).

(A) – Representative z-stacked immunofluorescence images showing histone-H1, myeloperoxidase (MPO) and DNA (sytox® and DAPI) on the surface of microcapsules. Polystyrene and PMMA microcapsules were explanted 3 days following implantation. Scale bar = 100 µm. In merged images, blue represents sytox, red – MPO and green – Histone-H1.

(B) – Representative z-stacked immunofluorescence images showing citrullinated histone-H3 and DNA (sytox® and DAPI) on the surface of microcapsules. PMMA microcapsules were explanted 3 days following implantation. Scale bar = 100 µm. In merged image, blue represents DAPI, green – cit-histone-H3 and purple – sytox. Data in 'B' and 'C' are representative of 1 independent experiment with n = 3 mice, and imaging of multiple microcapsules explanted from each mouse.

(C) – Measurement of neutrophil elastase activity in the peritoneal fluid of mock or alginate microcapsule implanted mice (2 weeks post implantation). * indicates $p<0.05$. Data are representative of 2 independent experiments with n = 6.
