# Supplementary material for: Neutrophil Responses to Sterile Implant Materials
Source: PLoS One. 2015 Sep 10;10(9):e0137550. doi: 10.1371/journal.pone.0137550 (PMC4565661; doi:10.1371/journal.pone.0137550)
Supplement: S1 Table — Endotoxin testing, culture of peritoneal fluid and general health of animals implanted with microcapsules suggested an absence of microbial contaminants or infections. (PDF) [file pone.0137550.s006.pdf]

Material	Testing Method	Results
Alginate Solution	Sold endotoxin free (below detectable limits) by manufacturer. Manufacturer's testing method unknown	-
Alginate microcapsules (prepared in our laboratory)	Limulus Amebocyte Lysate assay for endotoxins<br><br>Done in our laboratory<br>And Commercial testing at Charles River Laboratories	Below Detection Limits (< 0.05 EU / ml)
Saline (used to suspend microcapsules prior to implantation in mice)	Limulus Amebocyte Lysate assay for endotoxins<br><br>Done in our laboratory and Commercial testing at Charles River Laboratories	Below Detection Limits (< 0.05 EU / ml)
Glass microcapsules (purchased from commercial vendor)	Limulus Amebocyte Lysate assay for endotoxins<br><br>Commercial testing at Charles River Laboratories	Below Detection Limits (< 0.05 EU / ml)
Peritoneal Exudate (isolated from mice implanted with alginate microcapsules)	Limulus Amebocyte Lysate assay for endotoxins<br><br>Done in our laboratory	Below Detection Limits (< 0.05 EU / ml)
Peritoneal Cavity Fluid (swabbed from mice implanted with alginate microcapsules)	Swab culture by MIT division of comparative medicine core facility	No growth at 48 hours
Animals	Animal Health monitoring by MIT veterinary staff and regular weighing of mice	No weight loss at 2 weeks post implantation (supplementary fig. 4). Animals appear healthy according to MIT veterinary staff and MIT CAC guidelines

Supplementary Table 1: Absence of microbial contaminants and infections in the peritoneal cavity. Endotoxin testing, culture of peritoneal fluid and general health of animals implanted with microcapsules suggested an absence of microbial contaminants or infections.
